# Near-Field Electrospray ZnO Thin Film for Ultraviolet Photodetectors

**DOI:** 10.3390/mi17010069

**Published:** 2025-12-31

**Authors:** Liyun Zhuo, Tao Peng, Jiaxin Jiang, Gaofeng Zheng

**Affiliations:** 1 The Higher Educational Key Laboratory for Flexible Manufacturing Equipment Integration of Fujian Province, Xiamen Institute of Technology, Xiamen 361021, China; zhuoliyun@xit.edu.cn; 2 School of Mechanical, Electrical and Information Engineering, Xiamen Institute of Technology, Xiamen 361021, China; 3 School of Mechanical and Automotive Engineering, Xiamen University of Technology, Xiamen 361024, China; 4 Pen-Tung Sah Institute of Micro-Nano Science and Technology, Xiamen University, Xiamen 361102, China

**Keywords:** near-field electrospray, ultraviolet photodetector, ZnO thin film

## Abstract

ZnO thin-film ultraviolet photodetectors are widely used in the military, space, environmental protection, medicine, and other fields. Accurate printing of ZnO photoelectric-sensitive films plays a key role in the detection results. Therefore, obtaining printing technology with a simple process and high precision has become a challenge for ZnO photoelectrically sensitive films. By adjusting the distance between the nozzle and the collecting plate, the jet is atomized in a straight line and deposited directly on the collecting plate, which effectively improves the stability and controllability of the jet spraying and deposition processes. ZnO thin films with a uniform distribution of nanoparticles, significantly improved density, and controllable deposition area linewidth were successfully prepared. The effects of different ZnO film structures on the performance of ultraviolet photodetectors were tested. When the ultraviolet light intensity is 500, 1000, and 2500 mW/cm^2^, the $I_{light}$ of the photodetector is 4.62, 9.38, 14.67 mA, The on/off ratio ($I_{light}$/$I_{dark}$) is 20.7, 42.1, 65.8, implying satisfactory photoelectric performance as well as high stability and repeatability, providing an effective technical means for the precise printing application of micro-nano functional devices.

## 1. Introduction

An ultraviolet photodetector is a device that converts ultraviolet optical signals into electrical signals and is widely used in the military, space, environmental protection, medical, and other fields. With continuous research on semiconductor materials and device fabrication processes, the emergence of wide-bandgap semiconductor materials has provided new impetus for the research and application of high-performance ultraviolet detectors. ZnO is a direct wide-bandgap oxide semiconductor with a bandgap width of 3.37 eV. An exciton binding energy of 60 meV and high electron mobility result in a short carrier current transport time and fast response speed [1]. Research has found that ZnO ultraviolet photodetectors prepared using the wide bandgap characteristics of ZnO exhibit excellent response to ultraviolet light in both infrared and visible light backgrounds and have great potential for application in the field of ultraviolet photodetection [2]. Therefore, the precise printing and manufacturing of ZnO optoelectronic-sensitive thin films and the improvement of device structure integration have become the current research focus. Padha et al. [3] prepared a p-i-n type (p-Si/i-SiO_2_/n-ZnO) ultraviolet photodetector using low-temperature atomic layer deposition technology. Under a bias voltage of 0–18 V, the detector has a responsivity of 3.82 × 10^2^ A/W and a detectivity of 7.14 × 10^12^ Jones, with a rise time of 0.3 s and decay time of 0.2 s. Rajamanickam et al. [4] combined radio frequency magnetron sputtering technology, the hydrothermal method, and micro-nano processing technology to prepare Pd thin-film electrodes for ultraviolet photodetectors. Under a bias voltage of 1 V, the sensitivity and photoresponsivity of the detector prepared with the 1.7 mol% Ag-doped thin film were 105 and 0.4 A/W. Under a bias voltage of 5 V, the sensitivity and photoresponsivity of the detector prepared with the 5.4 mol% Ag-doped thin film were 32.91 and 2.1 A/W. Wang et al. [5] used the sol–gel method to prepare ZnO films and a hydrothermal method to prepare carbon quantum dots. They successfully prepared ZnO/CD hybrid photodetectors, which greatly improved the response speed of ZnO devices. For incident light at a wavelength of 365, 470, 515, 635, and 880 nm (23.08 mW/cm^2^) and voltage at 5 V, the detector responsivities are 3.70 × 10^−2^, 4.32 × 10^−5^, 1.80 × 10^−5^, 4.27 × 10^−8^, and 5.78 × 10^−9^ A/W, respectively. The detectivities are 1.08 × 10^10^, 4.91 × 10^7^, 1.63 × 10^7^, 3.32 × 10^4^, and 5.69 × 10^3^ Jones, respectively. Nguyen et al. [6] prepared a Schottky diode SiGe mid-wave infrared photodetector using photolithography technology that operates at room temperature with wavelengths between 5 and 8 μm and working pulses as short as 50 ns, exhibiting excellent detection performance. Prasad et al. [7] prepared high-performance photodetectors by growing single-layer WS_2_ thin films on silicon substrates through chemical vapor deposition, which exhibited high responsivity, high detection rate, and fast switching response time at specific light wavelengths. Although the above process methods can produce photo detectors with certain advantages, the process is relatively complex, and the cost is high. Electrospray is an additive manufacturing technology that mainly utilizes an external electric field to generate a large number of free charges on the surface of solution droplets, reducing the surface tension of the droplets. When the electric field force overcomes the surface tension, the suspended droplet undergoes liquid surface disturbance, stretching deformation, and forms a jet spray. Under the action of charge repulsion, it continuously ruptures, resulting in a large number of micro-and nanoscale droplets [8,9]. Electrospray is suitable for printing various materials such as polymers [10,11], semiconductors [12,13,14], metal particles [15,16,17], and ceramic materials [18] because of its simple equipment and good material compatibility. However, in traditional electrospray technology, the distance between the nozzle and the collection plate is relatively long, and the charged droplets undergo a long movement distance in space. Under the action of charge repulsion, a large deposition range will be formed on the collection plate, which makes it difficult to meet the application requirements of precise positioning and printing of micro-nano functional devices.

**Figure 1:**
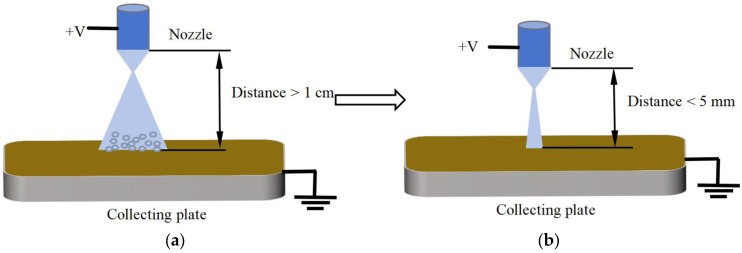
(**a**) Schematic diagram of traditional electrospray. (**b**) Schematic diagram of near-field electrospray.

Near-field electrospray technology is a new process that is based on traditional electrospraying. The core lies in optimizing the electric field distribution between the electrodes by shortening the distance between the nozzle and the collecting plate, ultimately achieving directional deposition control of charged micro and nanoparticles. In traditional electrospray experiments, a large distance is maintained between the nozzle and the collecting plate, with the printing principle illustrated in Figure 1a. This inevitably leads to the charged droplets being printed flying in the air for a longer period of time, with the droplets repelling each other due to carrying the same charge, thus forming a larger range of micro-nanoparticle deposition on the collecting plate. This enables the jet ejected from the Taylor cone to reach the collector almost instantaneously by reducing the distance between the nozzle and the collecting plate to the millimeter scale, thereby confining the deposition area within a range of several tens to hundreds of micrometers. After the spray printing deposition area is reduced, the patterned collection of micro nanoparticle films can be realized using a high-precision mobile platform to collect the deposited particles [19,20]. The spray printing principle is illustrated in Figure 1b.

Based on the near-field electrospray technology and the sensitivity of ZnO semiconductor nanoparticles to ultraviolet light, a ZnO thin-film-based ultraviolet photodetector was prepared, and the influence of the ZnO thin-film ultraviolet photodetector on the photoelectric response performance was analyzed.

## 2. Materials and Methods

### 2.1. Experimental Instruments and Equipment

**Figure 2:**
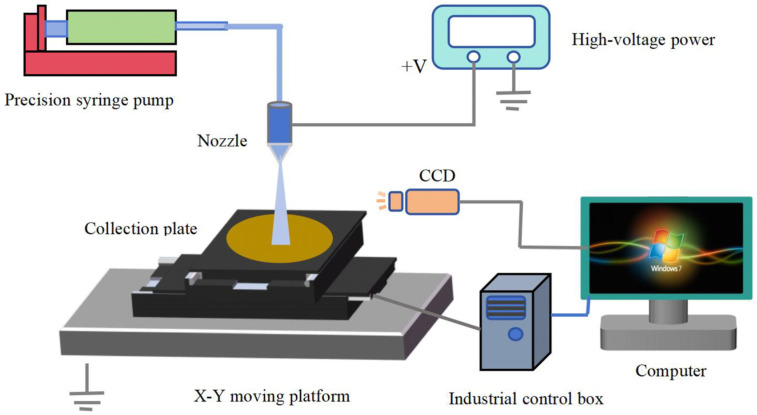
Near-field electrospray printing device.

The near-field electrospray experimental device is shown in Figure 2, which mainly consists of a precision injection pump, a nozzle, high-voltage power, a collecting plate, a computer, an X-Y moving platform, an industrial camera, and an industrial control box. The precision injection pump (Pump 11 Pico Plus Elite, Harvard Bioscience, Inc., Holliston, MA, USA) continuously supplies solution to the nozzle at a constant supply rate, the collecting plate is fixed on a precision two-dimensional motion platform (REI 95LM-050, Suzhou Gaohe Equipment Control Technology Co., Ltd., Suzhou, China), the nozzle uses a 32 G (inner diameter 0.10 mm) hollow dispensing needle, the positive pole of the high-voltage power (DW-P503-1ACH1, Tianjin Dongwen High Voltage Power Co., Ltd., Tianjin, China) is connected to the nozzle, and the negative output terminal is connected to the collecting plate and grounded, thereby forming a spatial high-voltage electric field between the nozzle and the collecting plate. The motion trajectory and speed were controlled using a computer and an industrial control box. The microfilm morphology was observed using a field-emission scanning electron microscope (SUPRA55 SAPPHIRE, Zeiss Optical Instruments, Oberkohen, Germany). The crystal structures of the as-prepared ZnO thin film were characterized through X-ray diffraction (XRD, Bruker D8-A25, Bruker AXS, Karlsruhe, Germany), and the solution rheology and jet spraying process were observed and recorded using a CCD (uEye UI-2250SE-C-HQ, IDS, Salbrucken, Germany). In this experimental device, the distance between the nozzle and the collection plate was adjustable from 0 to 5 mm. Under the action of the electric field force, the solution is stretched to form a “Taylor cone” at the jet outlet and finally stably sprayed from the tip. After being atomized in a straight-line segment, it is directly deposited on the collecting plate, and the predetermined patterned microstructure is obtained through the movement of the collecting plate. This overcomes the interference of charge repulsion and multi-stage rupture of charged droplets in the traditional electrospray by shortening the distance between the nozzle and the collecting plate, effectively improving the controllability of the jet spraying and deposition process, and greatly reducing the line width of the deposition area of the film.

### 2.2. Preparation Materials and Processes for Photodetectors

A nano-ZnO dispersion (50% aqueous solution, particle size 50 nm, Shanghai McLean Biochemical Technology Co., Ltd., Shanghai, China) was used as the experimental material to prepare the ZnO thin films. The contact electrode of the ultraviolet photodetector was a silicon-based interdigital electrode with an overall dimension of 7 × 5 × 0.5 mm. Monocrystalline Si was used as the electrode substrate. Silicon dioxide was thermally oxidized on the surface of a silicon wafer with a thickness of 285 nm. The metal layer structure was Ti/Au with a thickness of 50 nm/100 nm, 35 pairs of electrodes, an electrode spacing of 20 μm, and an electrode width of 20 μm.

**Figure 3:**
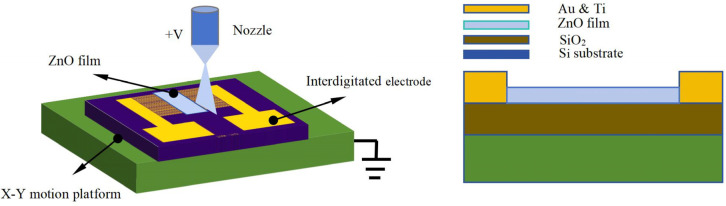
ZnO thin-film ultraviolet photodetector.

The ZnO thin-film ultraviolet photodetector prepared in this experiment is a metal-semiconductor-metal (MSM) type, which is composed of a double Schottky structure by combining a planar transverse interdigital electrode and semiconductor materials. The detector with this structure has the advantages of excellent response sensitivity, stability, and controllability, a simple process, and ease of realizing monolithic integration. The manufacturing process of the ultraviolet photodetector is as follows. First, the silicon-based interdigital electrode chip was cleaned with alcohol to remove dust and impurities on the surface of the electrode chip to avoid affecting the stability of the jet printing and the ultraviolet photoelectric detection effect. Then, the cleaned silicon-based interdigital electrode was tested to detect whether the chip electrode itself had an ultraviolet photoelectric response to avoid affecting the test effect of the ultraviolet photoelectric response of the subsequent spray-printed film. The silicon-based interdigital electrode chip was then placed on the moving platform, and the nozzle was adjusted to be above the silicon-based interdigital electrode chip through the sliding platform. Path planning of the moving platform was performed to ensure that the ZnO film could be deposited on the chip electrode. Finally, the near-field electrospray process parameters for the printing of the ZnO film layer were as follows: select a 30 G nozzle (inner diameter 0.15 mm), adjust the distance between the nozzle and the silicon-based interdigital electrode chip to 1 mm, move the speed of the receiving plate to 15 mm/s, and apply a voltage of 1.7 kV, with the positive voltage connected to the nozzle and the negative voltage connected to the receiving plate. The liquid supply speed was set to 160 μL/h, and the ZnO dispersion was removed after ultrasonic treatment with a syringe and installed on a precision syringe pump. The high-voltage and syringe pump were started, the spray printing solution was slowly advanced, the atomization spray printing jet was stable, the moving platform was started to move the planned path, and the spray printing of the ZnO film layer of the silicon-based interdigital electrode; the ZnO film ultraviolet photodetector with an MSM structure was obtained, as shown in Figure 3.

### 2.3. Optoelectronic Performance Testing of ZnO Thin-Film Ultraviolet Photodetectors

The working principle of the MSM ZnO thin-film ultraviolet photodetector prepared in this experiment mainly includes the processes of light absorption and photogenerated carrier generation, photogenerated carrier separation and transmission, and current formation and detection. Its specific working principle is as follows:

(1) Light absorption and the generation of photogenerated charge carriers.

ZnO is a wide band gap semiconductor material, and its band gap width is about 3.37 EV, which corresponds to the absorption of the ultraviolet band. When ultraviolet light irradiates the MSM-type ZnO Composite Film ultraviolet photodetector, the ZnO film absorbs the photon energy. When the photon energy is greater than the band gap width of ZnO, the electrons in the valence band absorb the photon energy and transition to the conduction band, leaving holes in the valence band and resulting in photogenerated electron–hole pairs, that is, photogenerated carriers.

(2) Separation and transmission of the photogenerated carriers.

In the MSM structure, the metal electrode and ZnO film form a Schottky contact and a Schottky junction. Under the action of an external bias, a built-in electric field was formed at the Schottky junction, and its direction pointed from the metal to the semiconductor. The photogenerated carriers are separated under the action of the built-in electric field in the Schottky junction; the electrons move in the negative direction, and the holes move in the positive direction. Because ZnO thin films have a certain crystal structure and energy band distribution, photogenerated carriers propagate along the crystal direction or defect states in the film. The distribution of ZnO particles in the ZnO film is more uniform, which is conducive to the transmission of photogenerated carriers between ZnO particles and reduces the recombination probability of the carriers.

(3) Current generation and detection.

After separation, the photogenerated electrons and holes moved to the corresponding metal electrode to form a photocurrent. In the external circuit, the magnitude of the photocurrent is related to the number of photogenerated carriers, the transmission efficiency of photogenerated carriers, and the structure of the detector. When light irradiation stops, the generation process of photogenerated carriers also stops, the generation of photogenerated carriers gradually compounds or becomes trapped, and the photocurrent gradually returns to the dark current level. The detection and response to ultraviolet light can be realized by detecting the change in current in the external circuit.

**Figure 4:**
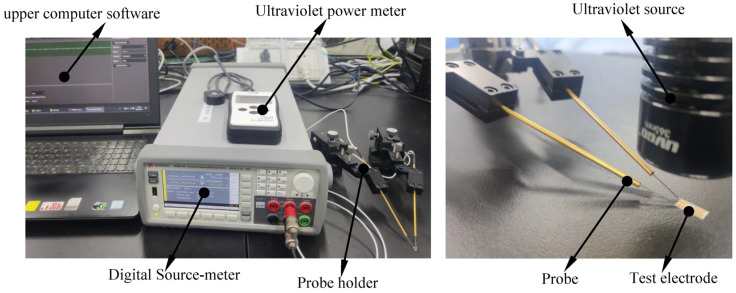
ZnO thin-film ultraviolet photodetector detection system.

In summary, MSM ZnO thin-film ultraviolet photodetectors generate photogenerated carriers through the absorption of ultraviolet light by ZnO, use the built-in electric field of the Schottky junction to realize the separation and transmission of carriers, and finally realize the detection of ultraviolet light by detecting the photocurrent. The ZnO film ultraviolet photodetector detection system built for this experiment is shown in Figure 4. Test process and method: To ensure the stability of the test, an ultraviolet photoelectric response test was carried out in a dark environment. In the early stages of the test, an ultraviolet lamp with a wavelength of 365 nm was used as the test light source. By adjusting the distance between the ultraviolet lamp and detector, the effect of different light intensities on the ZnO thin film was obtained. After each distance adjustment, the actual light intensity on the ZnO thin film was measured and recorded using an ultraviolet power meter. During the test, two probes were connected to both ends of the interdigital electrode, with the probes then connected to the positive and negative electrodes of the digital source meter through the probe base. After the bias voltage is applied at both ends of the interdigital electrode, the ammeter can detect the current signal generated in the photoelectric conversion process in real time and then obtain the *I*-*U*, *I*-*T*, and other current response characteristic curves of the photodetector.

## 3. Results

### 3.1. Preparation of ZnO Thin Films Using Near-Field Electrospray

**Figure 5:**
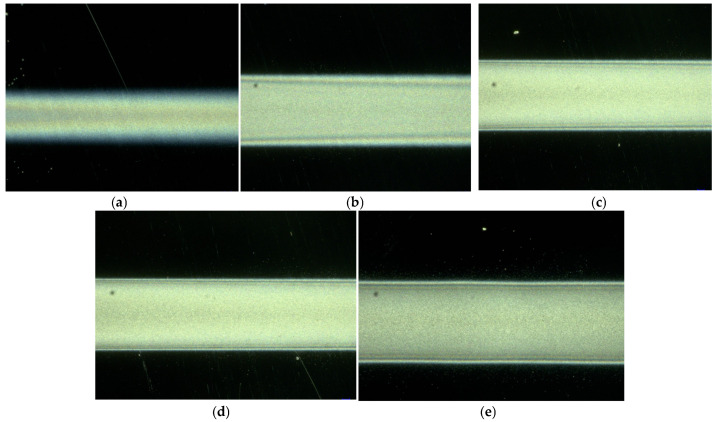
Morphology of ZnO thin film under different printing times. (**a**) one time; (**b**) three times; (**c**) six times; (**d**) nine times; (**e**) fifteen times.

**Figure 6:**
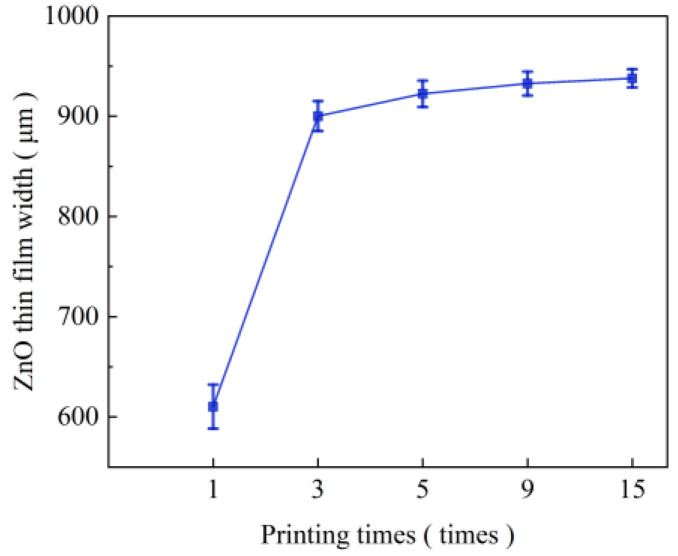
ZnO thin film width under different printing times.

In order to ensure the stability of the spray jet during the near-field electrospray, the liquid supply speed is set to 160 μL/h, the nozzle is selected as 32G (inner diameter 0.10 mm), the applied voltage is 1.7 kV, the distance from the nozzle to the receiving plate is 1 mm, and the moving speed of the receiving plate is 15 mm/s. The morphology of the near-field electrospray ZnO film for different spray times is shown in Figure 5. When the spray time was increased from one to three times, the particle density between the films increased significantly, and the distribution of nanoparticles was more uniform. This is because when the printing time is low, the distribution density of the nanoparticles in the film is lower, and the particles are distributed in the form of clusters in the ZnO film. The number of nanoparticles deposited on the collection plate per unit time was low, and the particles could not be tightly bonded. With an increase in printing time, the density of the nanoparticles increased significantly, and the nanoparticles could be tightly bonded and uniformly and continuously distributed throughout the film. At the same time, the increase in printing time also changes the film’s width. The relationship between film width and printing time is shown in Figure 6. The average width of the ZnO film increased from 610 μm to 900 μm when printing was performed one to three times. Although the width of the film was basically the same when the printing was increased from three to fifteen times, the density and distribution uniformity of the nanoparticles were improved.

**Figure 7:**
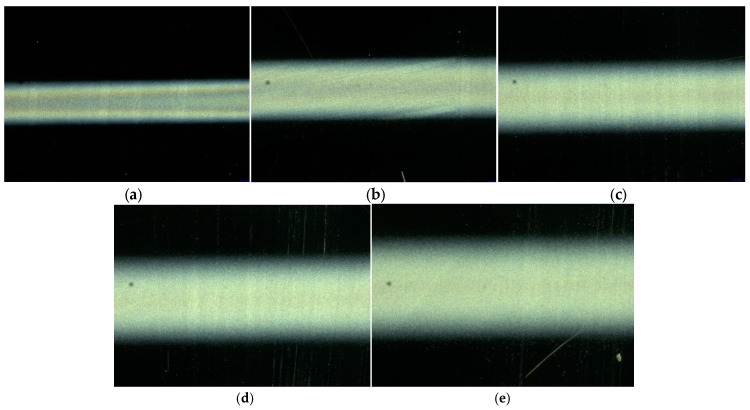
Morphology of ZnO thin films at different distances from the nozzle to the collecting plate. (**a**) 0.6 mm; (**b**) 0.8 mm; (**c**) 1.0 mm; (**d**) 1.2 mm; (**e**) 1.4 mm.

**Figure 8:**
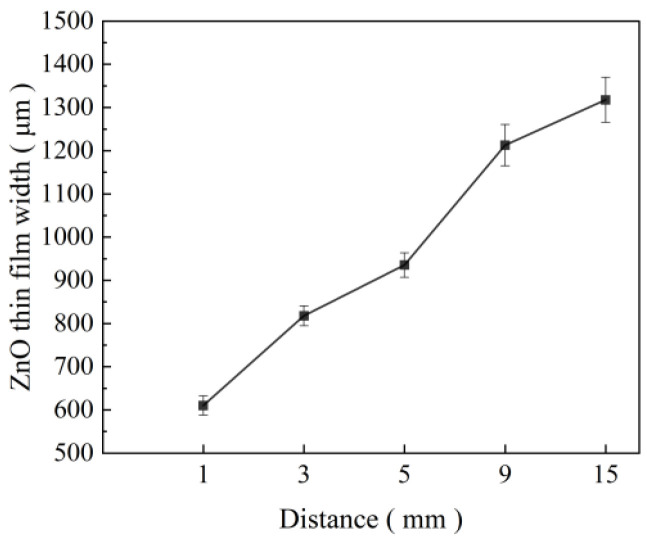
ZnO film width at different distances from the nozzle to the collecting plate.

To determine the influence of the receiving distance on the near-field electrospray printing results, the relationship between the distance from the nozzle to the collecting plate and the morphology of the ZnO film was explored by setting the nozzle at 32 G (inner diameter 0.10 mm), liquid supply speed at 160 μL/h, applied voltage at 1.7 kV, and moving speed of the receiving plate at 15 mm/s. The experimental results are presented in Figure 7 and Figure 8. As shown in Figure 7 and Figure 8, the width of the ZnO film increased with an increase in the distance from the nozzle to the collecting plate. When the distance is 0.6, 0.8, 1.0, 1.2, and 1.4 mm, the width of the printing film is 610.4, 818.0, 935.4, 1212.8, and 1317.8 μm, respectively. Even when the distance increases to 1.6 mm, the Taylor cone is unstable owing to the weakening of the electric field force, so the continuous atomized film cannot be sprayed because with an increase in the distance from the nozzle to the collecting plate, the electric field force gradually decreases, so the electric field force acting on the charged atomized particles in the space is weakened, and the particle trajectory is more dispersed, increasing the width of the sprayed film. Simultaneously, with an increase in the distance from the nozzle to the collecting plate, the charged atomized particles have more time to split and diffuse in the flight process, which increases the range of the printing area and further increases the film width. Simultaneously, as the receiving distance increased, the electric field force gradually weakened, and when it was less than the liquid tensile force, continuous jet printing appeared. In summary, the distance from the nozzle to the collecting plate is directly proportional to the width of the sprayed film. However, when the distance from the nozzle to the collecting plate increases to 1.6 mm, the spray printing state becomes unstable, and the ZnO film cannot be effectively sprayed and deposited.

### 3.2. Morphology of ZnO Thin Films

**Figure 9:**
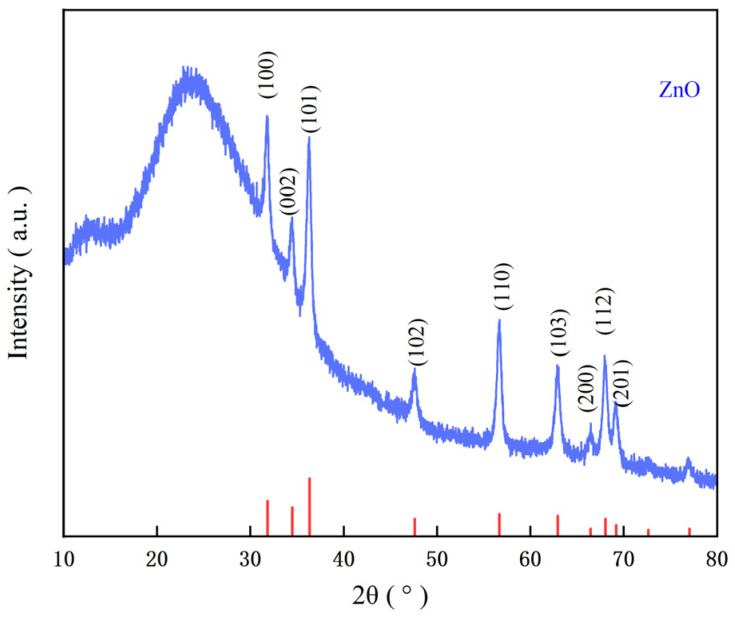
XRD patterns of the ZnO thin film.

**Figure 10:**
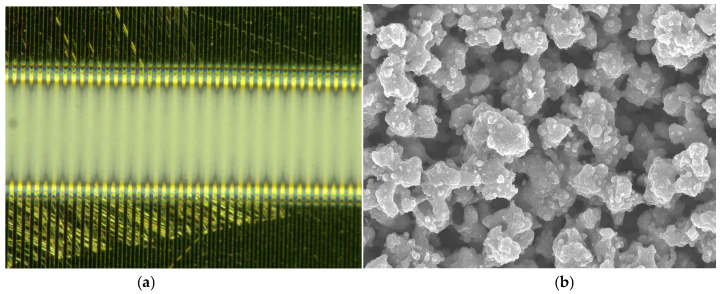
SEM image of the ZnO thin film. (**a**) ZnO thin film; (**b**) ZnO nanoparticles.

The XRD diagram of the ZnO film is shown in Figure 9. It can be found from Figure 10 that the diffraction peaks of the ZnO film at 2θ angles of 31.769°, 34.421°, 36.252°, 47.538°, 56.602°, 62.862°, 66.378°, 67.961°, and 69.098° correspond to the diffraction peaks of (100), (002), (101), (102), (110), (103), (200), (112), and (201) crystal planes of the ZnO material, respectively. It can be seen that the ZnO film we prepared is a polycrystalline film.

**Figure 11:**
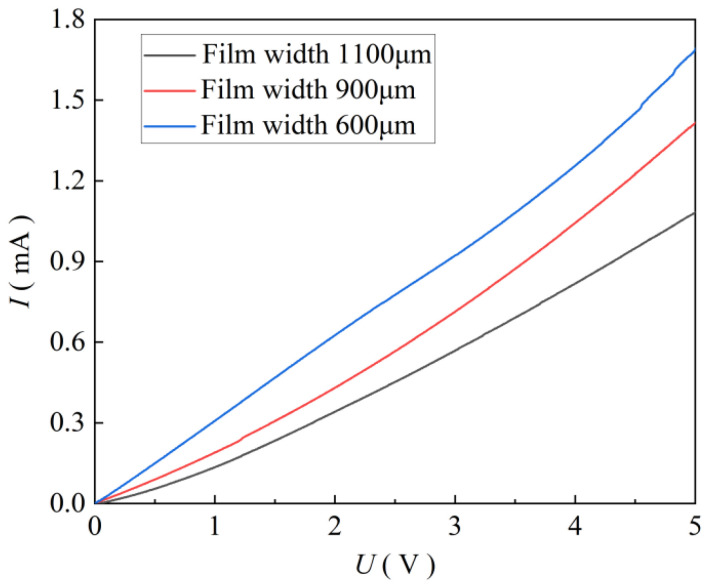
*I*-*U* curves at different film widths.

In order to better observe the morphology of the ZnO film, the surface of the film was tested by scanning electron microscope (SEM). Figure 11 is the SEM image of the ZnO thin film. It can be seen from Figure 10 that the ZnO film prepared by near-field electrospray has uniform distribution and good particle dispersion.

### 3.3. Photoelectric Performance Testing of ZnO Ultraviolet Photodetectors

To study the influence of the ZnO film width on the light current ($I_{light}$) of the detector, three types of photodetectors with film widths (600, 900, and 1100 μm) were prepared by changing the distance between the nozzle and the collecting plate and printing once. Under the condition of ultraviolet light intensity of 2500 mW/cm^2^, the $I_{light}$ of ZnO films with different widths were tested under an ultraviolet light intensity of 2500 mW/cm^2^. The measured *I*-*U* curves are shown in Figure 11. It can be seen from Figure 11 that the $I_{light}$ decrease with an increase in the ZnO film width. Under the condition of a bias voltage of 5 V, when the width of the ZnO film was 600, 900, and 1100 μm, the $I_{light}$ were 1.69, 1.42, 1.08 mA, respectively. This is because when the volume of the sprayed ZnO dispersion was constant, the smaller the width of the sprayed ZnO film, the more uniform the ZnO particles inside the film, the closer the bonding between the particles, and the better the carrier transmission efficiency, resulting in a greater light current.

**Figure 12:**
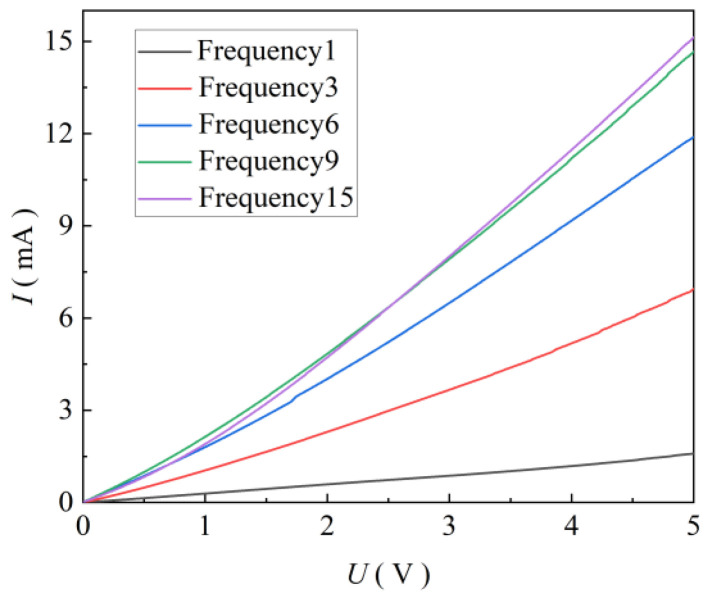
*I*-*U* curves of detectors at different printing times.

To determine the influence of different printing times on the lighting performance of ZnO thin-film ultraviolet photodetectors, the performance of ZnO ultraviolet photodetectors with different printing times was tested under an ultraviolet light intensity of 2500 mW/cm^2^. The *I*-*U* curve under ultraviolet light is shown in Figure 12. It can be observed from Figure 12 that the ($I_{light}$) increases with an increase in the number of printing cycles. When the bias voltage was 5 V and the printing times were one, three, six, nine, and fifteen cycles, the $I_{light}$ were 1.59, 6.95, 11.89, 14.69, and 15.13 mA, respectively. This is because, with the increase in printing time, the density of ZnO nanoparticles in the film is significantly increased, and the particles are closely bonded. The more uniform the distribution of nanoparticles in the film, the larger the transmission efficiency of photogenerated carriers, resulting in a stronger $I_{light}$ output. When the number of printing times reaches nine, the $I_{light}$ of the detector is basically unchanged when the number of printing times continues to increase. This is because the ZnO nanoparticles in the film have achieved uniform distribution, and continuing to increase the number of nanoparticles does not further enhance the photoelectric light current. Therefore, the ZnO thin-film UV photodetector with nine cycles of spray printing will be selected for the experiment.

**Figure 13:**
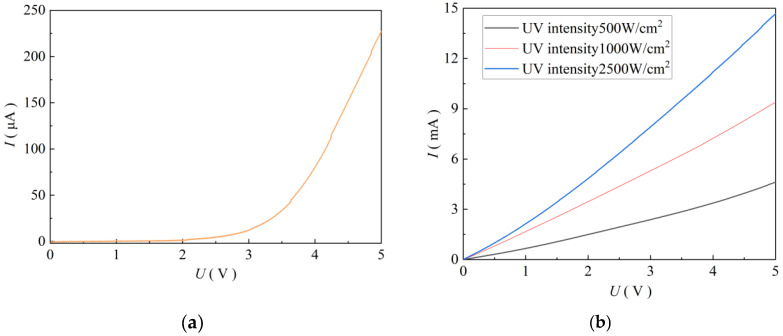
Current test under ultraviolet light irradiation. (**a**) No ultraviolet light; (**b**) Different ultraviolet intensities.

The response current of the ZnO thin-film ultraviolet photodetector printed nine times was tested with and without ultraviolet light. The results are shown in Figure 13a. It can be seen from Figure 13a that when there is no light, the dark current ($I_{dark}$) increases exponentially with an increase in the bias voltage, which indicates that there is an obvious potential barrier effect between the ZnO semiconductor film of the near-field electrospray in the sensor and the metal electrode on the interdigital electrode chip when there is no ultraviolet light. When the bias voltage is 5 V, the $I_{dark}$ is 223 μA. The results of the $I_{light}$ measured on the ZnO film under different ultraviolet light irradiations are shown in Figure 13b. As shown in Figure 13b, under ultraviolet irradiation, the $I_{light}$ increased significantly, and the size of the response current increased linearly with an increase in the bias voltage. When the bias voltage was 5 V, and the ultraviolet light intensities is 500, 1000, and 2500 mW/cm^2^, the $I_{light}$ of the photodetector is 4.62, 9.38, and 14.67 mA, respectively. The on/off ratio ($I_{light}$/$I_{dark}$) is 20.7, 42.1, 65.8, showing good photoelectric detection performance. This is because with the enhancement of ultraviolet light, more photons irradiate the ZnO film on the interdigital electrode chip per unit time, and the number of electron-hole pairs will increase, resulting in more carriers participating in the conductive process, thereby increasing the response current.

Responsivity (R) and detectivity ($D^{*}$) are important parameters of a UV detector, and the calculation formula is as follows:

(1) $$Ρ=\frac{{Ι}_{ph}}{PS}$$

(2) $$D^{*}=\frac{R}{\sqrt{2qJ_{d}}}$$

where $I_{ph}$ is photocurrent, $I_{ph}=I_{light}-I_{dark}$; P is the irradiation light power; S is the irradiation area; q is the amount of electric charge; and $J_{d}$ is the dark current density ($I_{dark}/S$). According to Formula (1), the responsivity of the ZnO UV photodetector is 209.37, 218.03, and 137.59 mA/W. According to Formula (2), the detectivity is 1.61 × 10^11^, 1.67 × 10^11^, and 1.06 × 10^11^ Jones, when the UV light intensity is 500, 1000, and 2500 mW/cm^2^, respectively. It can be seen that the ZnO UV photodetector prepared in this experiment has good response characteristics.

**Figure 14:**
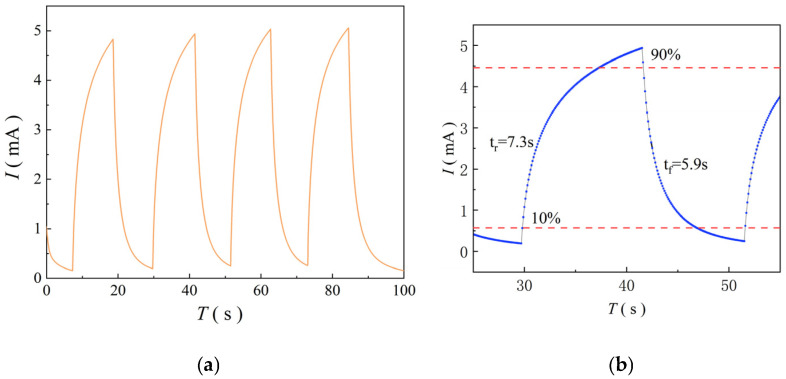
*I*-*T* curve of cyclic testing under light-dark conversion conditions. (**a**) *I*-*T* light-dark cycle conversion conditions; (**b**) *I*-*T* curve of a single cycle.

Under the condition of ultraviolet light intensity of 1000 mW/cm^2^ and bias voltage of 5 V, the ZnO UV photodetector was tested periodically in a light-dark conversion cycle experiment. The *I*-*T* curve of the test results is shown in Figure 14. It can be seen from Figure 14a that the ultraviolet ZnO photodetector prepared using near-field electrospray technology has good stability and repeatability. From Figure 14b, it can be seen that the rise time (10% to 90%) and fall time (90% to 10%) are 7.3 s and 5.9 s, respectively. After several light-dark cycle tests, ZnO UV photodetectors still show superior photoelectric response performance, which verifies the advantages of near-field electrostatic atomization technology in the preparation of high-performance UV photodetectors.

## 4. Conclusions

ZnO photosensitive thin films were prepared using near-field electrospray technology, and an MSM-type ZnO ultraviolet photodetector structure was developed. By adjusting the distance between the nozzle and the collection plate, the jet was directly deposited on the collection plate after being atomized in a straight-line segment, effectively improving the stability and controllability of the jet spraying and deposition process. It was found that by changing the number of printing cycles and adjusting the distance between the nozzle and the collection plate, ZnO thin films with a uniform distribution of nanoparticles, significantly increased density, and controllable deposition area and line width were obtained. The photoelectric performance of the different ZnO thin films was tested. When the ultraviolet light intensity was 500, 1000, and 2500 mW/cm^2^, the response photocurrent of the photodetector was 4.62, 9.38, and 14.67 mA. The on/off ratio ($I_{light}$/$I_{dark}$) is 20.7, 42.1, and 65.8, the responsivity of the ZnO UV photodetector is 209.37, 218.03, and 137.59 mA/W, and the detectivity is 1.61 × 10^11^, 1.67 × 10^11^, and 1.06 × 10^11^ Jones, showing good photoelectric detection performance. At the same time, through periodic light-dark conversion cycle experiments, the results show that the ultraviolet photodetector prepared by near-field electrospray technology has good stability and repeatability, with the rise time (10% to 90%) and fall time (90% to 10%) being 7.3s and 5.9s, respectively. After multiple light-dark cycle tests, it still exhibits superior photoelectric response performance, providing an effective technical means for the precise printing application of micro-nano functional devices.

## Data Availability

The data that support the findings of this study are available from the corresponding author upon reasonable request.
